# Adequacy of Usual Vitamin and Mineral Intake in Spanish Children and Adolescents: ENALIA Study

**DOI:** 10.3390/nu9020131

**Published:** 2017-02-13

**Authors:** Ana M. López-Sobaler, Aránzazu Aparicio, Liliana G. González-Rodríguez, Esther Cuadrado-Soto, Josefa Rubio, Victoria Marcos, Rosa Sanchidrián, Sara Santos, Napoleón Pérez-Farinós, Marian Ángeles Dal Re, Carmen Villar, Teresa Robledo, J. Javier Castrodeza, Rosa M. Ortega

**Affiliations:** 1 VALORNUT Research Group, Department of Nutrition, Faculty of Pharmacy, Complutense University, Plaza Ramón y Cajal s/n, Madrid 28040, Spain; araparic@ucm.es (A.A.); esther.cuadrado90@gmail.com (E.C.-S.); rortega@ucm.es (R.M.O.); 2 VALORNUT Research Group, Faculty of Health Sciences, Alfonso X El Sabio University, Villanueva de la Cañada, Madrid 28691, Spain; liligoro@uax.es; 3 Spanish Agency for Consumer Affairs, Food Safety and Nutrition, Ministry of Health, Social Services and Equality, C/Alcalá 56, Madrid 28071, Spain; jrubiom@msssi.es (J.R.); VMarcos@msssi.es (V.M.); rsanchidrian@msssi.es (R.S.); ssantos@msssi.es (S.S.); nperezf@msssi.es (N.P.-F.); mdalre@msssi.es (M.Á.D.-R.); cvillar@msssi.es (C.V.); trobledo@msssi.es, (T.R.); preaecosan@msssi.es (J.J.C.)

**Keywords:** usual intake, children, adolescents, micronutrient, vitamins, minerals, Spain

## Abstract

**Background:** The National Dietary Survey on the Child and Adolescent Population in Spain (ENALIA) provides data to assess the usual micronutrient intake among Spanish infants, children, and adolescents. **Methods:** Cross-sectional survey (November 2012–July 2014) of a representative sample of Spanish children and adolescents (six months–17 years) (*n* = 1862). Dietary information was collected using two non-consecutive one-day food diaries (six months–10 years old) or two 24 h dietary recalls (11 years and older) separated by at least 14 days. Estimates were calculated using the Iowa State University method and PC-SIDE software (version 1.0, department of statistics, center for agricultural and rural development, Ames, IA, USA) to account for within- and between-person variation. **Results:** Usual intake of vitamin D was insufficient in practically all individuals. Vitamin E, folate, and calcium were insufficient, especially from nine years of age, and magnesium and iodine from 14 years of age. The percentage of subjects with insufficient intakes was higher among females. Sodium intake was excessive in a considerable percentage of the population, especially in males, and it increased with age. Finally, over half of children under four years of age had zinc usual intakes that exceeded the Tolerable Upper Level. **Conclusion:** Vitamin and mineral intake in Spain should be improved, especially in late childhood and adolescence. Nutritional intervention and educational strategies are needed to promote healthy eating habits and correct micronutrient inadequacies in Spanish children and adolescents.

## 1. Introduction

Early childhood is the most rapid period of development and following a balanced diet in these stages is essential to ensure optimal growth and development [1]. Adolescence is another critical period of growth, during which the requirements for vitamins, minerals and trace elements increase substantially [2]. In addition, dietary habits are developed in childhood and adolescence and tend to remain through later stages of life [3,4,5], and unbalanced dietary habits during infancy and adolescence can have long-term implications, such as osteoporosis [6] and cardiovascular diseases [7]. This information highlights the importance of monitoring nutritional status and implementing health education programs in childhood and adolescence.

Dietary surveys provide insight into dietary habits and food intake and help estimate the adequacy of the nutrient intake of different groups in a population, monitor their changes and develop nutritional interventions. One of the challenges in dietary surveys is the assessment of usual intakes, i.e., the long-term dietary information. Single or repeated 24 h dietary recalls or food records are used for dietary assessment in large epidemiological studies [8] because they provide rich detail about food and beverage intakes. However, the within-person variation in short-term dietary data is large, so the intakes measured for a single day may provide a poor estimate of the long-term intake [9,10]. Repeated short-term measures are then needed, at least in a subgroup, to provide valid estimations for the population distributions of the usual dietary intake. Some methods have been developed for estimating the usual dietary intake based on repeated short-term measures [11], but few studies, especially in children, have applied them.

Until now the reference study on children and young people in Spain was the enKid study. It was conducted between 1998 and 2000 on 2855 Spanish children and adolescents aged between two and 24 years [12]. Dietary intake was assessed with one 24 h dietary recall and a second 24 h recall was used on a subsample (25%) to correct for the intra-individual variation to estimate usual nutrient intakes. Nutritional adequacy in Spanish children and adolescents was, in general, adequate, although high inadequate intakes of vitamins D, E, A, folate, iron, calcium, and magnesium were found. Adolescents were those with the highest nutritional risk, especially among girls. 

The ENALIA study (“Encuesta Nacional de ALimentación en Población Infantil y Adolescente de España” or “National Dietary Survey in Spanish Children and Adolescents”) is a cross-sectional survey that was carried out in Spain and sought to collect accurate food consumption data in children and adolescents in a way that is consistent with other European countries [13,14,15]. The ENALIA study was designed and developed by the AECOSAN (Spanish Agency for Consumer Affairs, Food Safety, and Nutrition) at the Ministry of Health, Social Services, and Equality to understand and monitor the diet of Spanish children and adolescents and to provide current data as a reference to analyze dietary trends over time. Thus, the present study provides recent data on the usual micronutrient intake from food and beverage sources for Spanish children, from six months until 18 years of age, and establishes the proportion of subjects who are most at risk for nutrient inadequacy and/or adverse effects.

## 2. Materials and Methods

### 2.1. Study Design

The ENALIA study was a cross-sectional survey conducted in Spain on a national sample of children and adolescents (six months–18 years old). The design, protocol, and methodology of the ENALIA study have been already described elsewhere [16,17] and followed the European Food Safety Agency (EFSA) “EU Menu” guidance recommendations [15]. This survey had a stratified cluster-sampling design and was representative at a national level. Briefly, the ENALIA study was designed to collect food consumption data and information on eating habits in Spanish children and adolescents between six months and 18 years of age. 

The study was conducted according to the guidelines laid down in the Declaration of Helsinki. Depending on the age of participants, information was given to parents, tutors, or other legal representatives, and consent and assent from each participant were obtained before proceeding with the study interviews. The Spanish Agency of Consumption, Food Security, and Nutrition (AECOSAN), belonging to the Spanish Ministry of Health, Social Services and Equality, approved the study. In accordance with the Spanish Ethical Review System, ethical approval was exempt from ethical review because this is a population-based survey, no intervention were done, no human biological samples were collected, and all data from the study were anonymized and de-identified.

### 2.2. Sample

The target population was people under 18 years old living in households in Spain. The ENALIA study began in November 2012 and ended in July 2014. Institutionalized populations were excluded. Recruitment was done via home visits and for children under four years of age, also in kindergartens, since in Spain they are only for the day care of the children. Subjects were randomly chosen from all 17 autonomous regions in Spain, according to a random multistage sampling procedure, considering census section and municipality, population size (<10,000, 10,000–100,000, 100,000–500,000, >500,000 inhabitants), age, and sex. To capture the inter-seasonal variability in consumption patterns, subjects were uniformly distributed over the four different seasons, and the schedule was organized to capture an adequate proportion of weekdays and weekend days at the population group level. The sample was also distributed uniformly over the weeks in a month. The recruitment process included five contacts: an initial contact with household by phone or at nurseries/kindergarten (for children under three years of age), a second contact by letter (including the general questionnaire and the diary/24 h recall), two more contacts by phone (to confirm the consent to participate, to conduct the general questionnaire in the third contact, and the first diary/24 h recall in the fourth contact), and a fifth final contact with a household visit (where the second diary/24 h recall and anthropometric study were done).

The ENALIA final sample included 1862 children aged six months to 17.9 years. The participation rate was 68.9%. Table 1 describes the sample size by age and gender. 

### 2.3. Dietary Study

Dietary information was collected using two different methodologies depending on the child’s age: two non-consecutive one-day food diaries separated by at least 14 days, for children from six months to 10 years of age or two 24 h dietary recalls, separated by at least 14 days, for adolescents (11 years old and over). In younger groups of children (under 11 years old), parents and caregivers were responsible for completing the food diaries and the other questionnaires, and requested collaboration and/or information from other proxy persons about the child’s out-of-home diet. The recalls were conducted using specific software (ENIA-Soft) (version 5.0, Demométrica SL, Madrid, Spain) in computer-assisted interviews by trained interviewers and nutritionists/dieticians. Food diaries were also recorded using this software, since guided the interviewer during data collection, facilitating the homogenous collection of information. The software included a database with coded foods, a database with standard recipes, a database with weights and household measurements, and a database with pictures and portion weights from a validated photographic food atlas for Spanish children and adolescents [16]. The pictures were used for the correct identification of the dishes, which were then disaggregated into their ingredients according to standard recipes. It was possible to adapt standard recipes to the actual ingredients used (including salt). Consumed amounts were estimated using the pictures complemented by household measures and portions indicated in standard recipes. For both food records and 24 h recalls participants were asked about the use of salt at each eating occasion (during preparation, cooking and at the table), and the type of salt consumed (regular or iodized). A food propensity questionnaire (which included the frequency of use of supplements) that was specially designed for infants and adolescents complemented both methods [16]. Of the 1862 children, 1780 provided two dietary records/recalls.

Nutrient intake was calculated from dietary records/recalls using the Spanish Food Composition Tables [18] completed with additional data on nutrient composition for specific brands and enriched/fortified foods. There was only information on the frequency of use of supplements, not on quantities and brands used, so any data on nutrient intake represented only that from food and beverages. Niacin was expressed as equivalents of niacin (preformed niacin + tryptophan/60). For vitamin A from β-carotene, a conversion factor of 1/6 was used, whereas for the other carotenoids, a conversion factor of 1/12 was used. Vitamin E was expressed as alpha-tocopherol equivalents, and folate intake was calculated as µg of dietary folate equivalents (DFE) (food folate + 1.7 × synthetic folic acid content of fortified food).

### 2.4. Socio-Demographic and Anthropometric Information

A general questionnaire asked about sociodemographic data, such as the birth date of the participant, place/country of origin of the participant and his/her parents, academic level and profession of parents, and about the health status of the participant (e.g., special diet, use of drugs, chronic or acute diseases).

Anthropometric data of the participant were measured at the interviewee’s home by trained interviewers and following standardized procedures [19]. A stadiometer was used to measure stature in subjects aged two years and older, and an infantometer was used to measure the recumbent length of subjects aged six to 24 months old. Stature and length were measured in meters. A digital weight scale with an accuracy of 0.1 kg was used. 

### 2.5. Statistical Analysis

Sample weight factors for each participant were calculated to account for nonresponses and to weight the sample to known population demographic characteristics. Since the average intake of a small number of days of dietary record/recall (observed intakes) does not adequately reflect the usual intake, it is necessary to statistically model the dietary data to eliminate day-to-day variation in food consumption [12]. We used the method developed by Nusser et al. [20], also known as the Iowa State University (ISU) method. Briefly, the ISU method consists of three steps: (1) a transformation step that maps intakes into a normal scale; (2) an estimation step to estimate the parameters of usual intake by using a measurement error framework; and (3) a back-transformation step to return the estimated usual intakes to their original scale. The ISU method was implemented using the PC-SIDE software (version 1.0, 2003), which was designed for this purpose. This program estimated the percentiles of usual nutrient intake distributions and the proportions above or below the defined Dietary Reference Intakes (DRI) cut-off values. The interview day (day 1 or day 2), the day of the week, season, and sample weighting factor were considered in the adjustment of dietary data, stratifying by sex and age group.

To assess vitamin and mineral adequacy, the Estimated Average Requirement (EAR) cut-off point method was used [21]. We used the Institute of Medicine’s (IOM) DRIs since the Spanish DRIs do not provide estimated average requirement (EAR) values we required for our analyses. IOM’s DRI are regularly updated and frequently used compared to reference values provided by other scientific bodies of organizations. The proportion of the population with usual intakes less than the EAR provides an estimate of the proportion of the group whose intakes do not meet the nutrient requirement. There are only established EARs for iron and zinc for children under one year of age. The Adequate Intake (AI) cannot be used to determine the proportion of individuals in a group with an inadequate nutrient intake. For Fe, we used the probability approach recommended by the Institute of Medicine [22] because the presence of menstruating women skews the requirement distribution curve and renders the EAR cut-off point inappropriate. For nutrients with established Tolerable Upper Intake Levels (ULs), the proportion of children with usual intakes exceeding the UL were computed. 

The plausibility of energy intakes was assessed using the Goldberg’s cut-off method [23] updated by Black [24], following the methodology applied in other European children [25]. Basal metabolic rate (BMR) was estimated by Schofield equations [26], taking into account age, sex, body height, and weight [23,24]. Age- and sex-specific cut-offs for children and adolescents were calculated as suggested [27], using specific reference values for the within-subject coefficient of variation for energy intake (EI), BMR, and physical activity, as given in Nelson et al. [28] and Black [24]. Under reporters were identified as those with EI/BMR ratios up to 0.73–1.08, while over reporters were identified by EI/BMR ratios above 2.29–2.88, depending on the subject’s age and sex.

Statistical analyses were performed using the statistical software package SPSS version 20.0 for Windows. A *p* value < 0.05 indicated statistical significance. The normality of the distribution of age, anthropometric and EI/BMR data was checked using the Kolmogorov–Smirnov test. Differences between males and females were examined with the Student’s *t* test or the Mann-Whitney U test, depending on whether or not the data were normally distributed. Categorical variables were compared using the χ^2^ test. Dietary intakes of the participants were expressed as the means and standard deviations, medians, and 5th and 95th percentiles.

## 3. Results

Characteristics of the studied sample are shown in Table 1. The highest percentage of users of dietary supplements was found in the group of children between six and 12 months (24.7%). The percentage of users in other age groups ranged from 3.6% (in children between four and eight years) to 7.7% (14–18 years). The most used supplement in children between six and 12 months was vitamin D (20.3% of children), while only 1.5% of children between one and three years used it. In the other age groups, less than 0.5% of children used vitamin D supplements. Regarding multivitamins with or without minerals, their use ranged from 1.0% in children between six and 12 months and 3.4% in children between nine and 13 years.

The percentage of under-reporters ranged from 0.6% (children aged six to 12 months) to 19.8% (adolescents aged 14–17 years). On the other hand, overestimation was higher in younger children (16%) and lower in adolescents (0.4%). The data presented in the rest of this report have not been adjusted for under-reporting.

The usual intakes (from food and beverage sources only) of vitamins and minerals are presented in Table 2 and Table 3, respectively. Dietary inadequacy regarding vitamins was particularly high for vitamin D, which was inadequate in almost all of the population, followed by vitamin E, especially in adolescents over 14 years. Water-soluble vitamin intakes were adequate in general, except for folate, because more than 50% of the participants over nine years of age had intakes below the specific sex-age EAR. Vitamins A, C, and thiamin intakes (in females) were also inadequate in a subset of adolescents (10.1%–13.0%, 7.8%–5.4% and 8.2%, respectively). The vitamin intakes did not exceed the ULs, except that of folate in a subset of children between one and three years of age (4.4%–6.1%, Table 2).

Regarding the usual intake of minerals, the prevalence of inadequate intakes in children below four years of age was low (<5%), except for iron in children between six and 12 months and for iodine in children between one and three years, where a subset of infants (10.9%–14.2% for iron and 11.0%–7.8% for iodine, Table 3) had inadequate intakes. The prevalence of mineral inadequacy in older children and adolescents was in general very low, except for those of calcium (especially in children over nine years of age), magnesium and iodine (in adolescents 14 years old and over). Mineral inadequacy tended to be higher in adolescent females. There are no established EARs for potassium, but the median and 95th percentile intakes suggest that a low percentage of the participants had suitable potassium intakes.

The usual intake of minerals exceeded the ULs for sodium in a high percentage of children and adolescents, and the prevalence of higher usual intakes increased with age (Table 3). In addition, more than half of the population had usual zinc intakes that exceeded the ULs in children between six months and four years of age.

## 4. Discussion

This paper provides recent estimates of the vitamin, mineral, and trace element intake distributions, from food and beverage sources only, in a representative sample of Spanish infants, toddlers, children, and adolescents. The usual micronutrient intakes in the ENALIA study meet or exceed their requirements, except for those of vitamin D, which was practically insufficient in all individuals, and vitamin E, folate, calcium, magnesium, and iodine, especially in adolescents and females. Sodium intake is excessive in a considerable percentage of the population, especially in males, and increases with age. Finally, the usual intake of zinc exceeds the UL in children less than four years of age.

Data on intake distributions in children, corrected for the day-to-day variation, are very scarce in Europe [29]. As for studies in Spain, the enKid study, conducted between 1998 and 2000, has been the reference study until now. Although the intakes were adjusted for random intra-individual variation, the proportion of the population that is at risk for deficits was estimated, using the recommended intake (RI) for the Spanish population and/or one-third and two-thirds of the RI as cut-off points [12]. The key findings were that vitamins A, D, E, folate, iron, calcium, and magnesium were the nutrients most likely to be associated with inadequate intakes, and that adolescents aged between 14 and 17 years were the group with the highest nutritional risk, especially among girls. Due to the methodology followed, the enKid study could not assess the risk of excessive intakes. In this sense, the ENALIA study provides current data on the usual intakes from food and beverage sources and the risk of inadequate intakes in Spanish children and adolescents using the EAR and the UL as the IOM recommends [21]. 

Both food diaries and dietary recalls are prone to under-/over-reporting, and there is debate about whether or not to eliminate misreporters in dietary studies, since the exclusion of potential misreporters may create a biased sample. The exclusion of under-/over-reporters might have induced selection bias since the misreporters might have a special food choice or eating behavior. In addition, under-reporting includes both under-recording and under-eating, and some over-reporters could eat much more than usual during the period of study as well. It has also been suggested that low energy reporting may be just as common among plausible energy reporters as among those defined as under-reporters [30], so that selectively excluding those with implausible energy intakes could bias the results. In addition, during childhood, diet tends to be highly variable from day to day and the identification of under-reporters is difficult [31,32]. Therefore, and following EFSA recommendations [33], we did not exclude potential misreporters from the analysis.

This study highlights the very low intake of vitamin D, which is insufficient in all age groups and sexes, and the fact that almost all individuals have intakes that are at risk. Vitamin D deficiency is a worldwide health problem, and similar data have been described in infants in the Netherlands [34], Finland [32], and Belgium [35], and in European adolescents [36]. The high prevalence of inadequate intakes may be justified by the fact that vitamin D is found naturally only in a few foods, and even food fortification has a modest effect on vitamin D status [37]. Vitamin D is found naturally in fish, egg yolk, and offal, such as liver [38]. Typical fortified foods are milk, breakfast cereals, and margarine [39]. However, consumption of these foods (eggs, fish, cereals, and dairy) is low in a significant percentage of Spanish children and adolescents [40]. Although we could not assess the risk of inadequacy or excessive intake of vitamin D in children between six and 12 months, we must consider that 20% of the children in the group used vitamin D supplements. In addition, although the use of multivitamin supplements was much lower, it may also contribute to the intake of vitamin D. On the other hand, less than 1% of children older than 12 months used vitamin D supplements and less than 7% used multivitamin supplements that could also contribute to the intake of this vitamin. Taking this into account, we can concluded that, in spite of the lack of data on the supplements, the risk of insufficiency is high in all children, with those between six and 12 months being at the lowest risk. Additionally, although vitamin D can be synthesized by sunlight, sun exposure is not always sufficient to offset the low dietary intake [41]. In fact, the serum levels of vitamin D are insufficient in Spanish schoolchildren, since 47% had hypovitaminosis, and 35% had a clear deficiency in vitamin D [42].

The usual mean vitamin E intakes were less than the EARs in some subgroups of children, especially those over nine years of age, similar to what was observed in other toddlers and preschoolers [35] and in adolescents in the Healthy Lifestyle in Europe by Nutrition in Adolescence (HELENA) study [36]. Vitamin E deficiency is rarely found in adults, but is more frequently found in children, likely because they have limited stores and are growing rapidly, thereby allowing such deficiency symptoms to be readily apparent [43]. A recent systematic review concluded that vitamin E intake was insufficient in 61% of the reviewed studies and that 13% of the subjects, mainly newborns and children, were below the functional deficiency threshold for vitamin E, which suggests that the α-tocopherol status is inadequate in a substantial part of the studied populations [44]. 

Folate intake is inadequate in a high proportion of children and adolescents over nine years of age (Table 2). The risk of inadequacy is higher in adolescent females. Similar results have been shown in other studies in adolescents [36,45]. Folate is essential for optimal growth, development, and maintenance of health throughout all life stages. Adequate folate status among reproductive-aged women is critical, given the important biological role of folate in gene expression, cell division, and reproduction [2]. Folate is important not only for the prevention of neural tube defects during pregnancy but also for the prevention of cardiovascular disease (CVD) and some malignancies [46]. Thus, the low folate intake among Spanish adolescents is of great concern.

Regarding minerals, the risk of inadequate calcium intake is especially high in children over nine years of age and is even higher in females (Table 3). Diethelm et al. [36] showed low intakes in European adolescents in the HELENA study, and Ortega et al. [47] found low intakes of calcium in a representative sample of Spanish schoolchildren between seven and 11 years of age. Although it is an essential nutrient, there is controversy about whether calcium intake in the population is high or insufficient [48]. The same goes for the consumption of dairy products [49], which are the main source of this mineral [47]. While some authors have suggested that the intake of dairy products is high in Spanish children [50], others have indicated that it is insufficient and can clearly be improved [47,51]. Some authors indicated that their consumption may be associated with an increased risk of some cancers, such as prostate and ovary [52], and even bone fractures [53], but other studies suggested that high intakes of calcium probably protect against colorectal cancer, bladder cancer, gastric cancer, and breast cancer, and that dairy intake does not seem to be associated with the risk of pancreatic cancer, ovarian cancer, or lung cancer, whereas the evidence for prostate cancer risk is inconsistent [54]. In addition, the evidence confirms that milk and dairy intake has a positive effect on bone health in childhood and adolescence, decreases the risk of childhood obesity, improves body composition in adults, and contributes to lower risk of developing type 2 diabetes [54].

As for iron intake, the EARs are exceeded in almost all subgroups, except for a small but important subset of six- to 12-month-old infants whose usual intakes were below the EAR (10.9% in males, 14.2% in females, Table 3). These figures correspond only to iron intake from foods and beverages, and since the use of iron supplements/multivitamins with minerals that could contain iron was very low in this age group, it is unlikely that the use of supplements had a significant impact on iron intake. These data are similar to those found in recently in the USA [55] and Spain [56], and could be consistent with the figures for prevalence of iron deficiency (9.6%) and iron deficiency anemia (4.3%) found in children below 12 months in Spain [57]. Since iron deficiency during early years may interfere with cognitive development [58], it is necessary to pay more attention to this problem.

On the other hand, the percentage of adolescent girls with inadequate iron intake is low relative to other studies, although differences may be due not only to different eating patterns of children and adolescents within Europe [59], but also to methodological differences. For example, the enKid study [12] found 26% and 34% of girls between 10–13 years and 14–17 years, respectively, had intakes lower than two-thirds of the AI (AI = 18 mg/day). This cut-off point is close to the EAR established for children between nine and 13 years, but is lower than that the EAR for adolescents between 14–17 years. Data from the HELENA study in European adolescents [60] found 3% of insufficient intakes in boys and 12.2% in girls between 12.5 and 17.5 years. However, this study assumed that all girls were menstruating, which may have slightly overestimated the percentage of girls below the EAR in the younger girls. Additionally, the EAR cut-point method was used for estimating the prevalence of inadequate intakes, and this may produce an underestimation of the true prevalence of inadequate intakes among girls.

The usual intake of sodium exceeds the UL in a considerable percentage of children in all age and sex groups, although the prevalence of excessive intakes is higher in males (Table 3). Similar results have been shown in other groups of preschoolers [35] and European adolescents [36]. In addition, the median intake of potassium is below the AI. Some studies indicate that elevated blood pressure, which may lead to stroke, CVD, and kidney disease, is associated with increased sodium and inadequate potassium intake [61]. Consequently, these increased sodium intakes and inadequate potassium intakes are a cause of concern for Spanish children and adolescents. We must bear in mind the inability to precisely account for added salt, so it is likely that sodium intake is even greater than that recorded. The best method for estimating sodium intake is the measurement of its excretion over 24 h, since dietary surveys and databases detailing food composition underestimate sodium intake. In this sense, the Spanish Food Composition Tables [18] include data from both analytical and bibliographic sources, as well as information provided by the manufacturers. The latest update has been made on the sodium data, based on analytical data of more than 1000 brands of processed foods (snacks, biscuits, preserved vegetables, fish and meat, sausages, prepared dishes, etc.). On the other hand, data on sodium excreted in 24 h urine from 205 Spanish schoolchildren aged between seven and 11 years have recently been published [62]. The mean 24 h urinary excretion of sodium was 132.7 ± 51.4 mmol/24 h, equivalent to 3052 ± 1182 mg Na/24 h (salt equivalent: 7.8 ± 3.1 g/day). Moreover, 84.5% of subjects aged ≤10 years had intakes of >4 g of salt per day, and 66.7% of those aged >10 years had intakes of >5 g salt per day. This is in line with our dietary results, which, although subject to error, confirm the need to take measures to reduce salt intake in Spanish children and adolescents. Previous studies indicated that the main sources of sodium in Spanish schoolchildren were added salt (at table or cooking) (21.3%), chip potatoes (12.1%), bread (11.3%), and cured and cooked ham [50]. Therefore, it seems prudent to reduce the consumption of processed products, and to promote the consumption of non-processed fresh foods with low sodium content.

The usual magnesium intake is insufficient in 63.5% of adolescent males and 81.8% of females (Table 3). The median intake in the adolescent group is similar to that reported in the HELENA study [36]. Accumulating evidence supports the notion that magnesium plays significant roles in promoting strength and cardiorespiratory function [63], and an inverse association between dietary magnesium and metabolic syndrome and its components has been shown [64].

Some small but important subsets of children have a high risk of inadequate intake of iodine, and the risk is highest in adolescent females over 14 years (Table 3). Other studies have also found inadequate intakes of iodine in other groups of adolescents [36,59]. It is difficult to estimate the intake of iodine because much of the intake of this mineral comes from iodized table salt. Although we considered the use of table salt in our study and the subjects were asked about the type of salt used, it is possible that the use of iodized salt has not been properly determined. 

Finally, the usual zinc intake exceeds the UL in children up to four years of age, although there was a group of girls over nine years old with intakes at risk of deficiency (9.2% and 6.3% in girls between nine and 13 years and over 14 years, respectively, Table 3). Goldbohm et al. [34] observed that 17% of children aged 10 to 48 months in the Netherlands have excessive intakes of zinc, and Butte et al. [65] described a similar situation in toddlers and preschoolers in the USA, which is justified by the frequent use of fortified foods containing zinc such as infant formula, infant cereals, and ready-to-eat cereals. We must keep in mind, however, that the validity of the UL for zinc for infants and children aged one to three years has been questioned based on the paucity of data used to establish the UL and the absence of zinc toxicity with usual zinc intakes in the US pediatric population [65]. 

Our study has some limitations and strengths. First, the food composition tables may not accurately reflect the nutrient composition of the food. It is appreciated that there is variability in the composition of foods, likely due to differences in season, cultivar, or variety. On the other hand, the Spanish Food Composition Tables used included enriched/fortified foods commonly available in Spain, and additional composition data for specific brands were taken into account. Fortification values can differ between countries, so data from food composition tables from other countries might not be adequate. Our study did not account for the use of supplements of vitamins and minerals, so the results of this study are limited to the dietary intake of micronutrients. Although parents may be reliable reporters of their children’s food intake at home, meals out of parental control are prone to being misreported, as are the estimation of their portion sizes [66]. Finally, causality for dietary intake not meeting the dietary guidelines cannot be confirmed due to the lack of biochemical data and functional parameters. On the other hand, one of the major strengths of the present study, in addition to its large sample size, is its representativeness of the population and the use of standardized and validated procedures. The 24 h dietary record in children and 24 h dietary recalls in adolescents provide rich detail about the types and amounts of food consumed and were shown to be less prone to systematic bias compared with food frequency questionnaires [67]. Application of the ISU method corrected the data for day-to-day variation, although we should still bear in mind that the true intake distributions remain unknown because of the lack of objective validation data. The assessment period in our study covered an entire year, considering seasonal variations in diet, and because all days of the week were included in the study, the effects of the day of the week could be removed. 

## 5. Conclusions

This paper provides recent data on the dietary vitamin and mineral intake from food and beverage sources of Spanish children. Public health initiatives should ensure that children and adolescents follow an adequate, balanced diet because the health behaviors adopted during the early years of life and adolescence can be maintained in adulthood, and improving them would help prevent many chronic diseases related to diet. Such initiatives should include the promotion of a Mediterranean Diet style, including more fish, eggs, cereals, and dairy products which are source of vitamin D, and lowering the consumption of processed foods which are sources of sodium. Additionally, the collaboration between the administration and the industry aiming to reduce more of the sodium content of processed foods is also desirable. Clinical and biological assessment of the nutritional status of a representative sample of Spanish children and adolescents focusing on the micronutrients of concern would seem advisable.

## Figures and Tables

**Table 1:** Characteristics of the studied sample in the ENALIA study.

	Total	Boys	Girls
*n*	1862	967	895
Age (years), X ± SD	8.8 ± 4.9	8.9 ± 4.9	8.8 ± 4.8
**Age group, *n* (%)**			
6–12 months	292 (15.7)	138 (14.3)	154 (17.2)
1–3 years	407 (21.9)	218 (22.5)	189 (21.1)
4–8 years	418 (22.5)	211 (21.8)	207 (23.1)
9–13 years	470 (25.2)	243 (25.1)	227 (25.4)
14–17 years	275 (14.8)	157 (16.2)	118 (13.2)
**Anthropometric characteristics**			
Weight (kg), X ± SD	34.25 ± 18.15	35.36 ± 19.23	33.07 ± 16.88 *
Height (cm), X ± SD	131.6 ± 30.4	133.0 ± 31.5	130.1 ± 29.1 *
BMI (kg/m^2^), X ± SD	18.1 ± 3.1	18.1 ± 3.0	18.0 ± 3.1
Z-BMI, X ± SD	0.33 ± 1.20	0.37 ± 1.27	0.29 ± 1.13
**Community** **size ^a^*n*, (%)**			
<10,000	358 (19.2)	184 (19.0)	174 (19.4)
10,000–100,000	761 (40.9)	380 (39.3)	381 (42.6)
100,000–500,000	466 (25.0)	256 (26.5)	210 (23.5)
>500,000	277 (14.9)	147 (15.2)	130 (14.5)
**Father’s education (%)**			
Mandatory or less ^b^	573 (31.1)	300 (31.4)	273 (30.8)
Secondary	536 (29.1)	283 (29.7)	253 (28.6)
University	731 (39.7)	371 (38.9)	360 (40.6)
**Mother’s education (%)**			
Mandatory or less ^b^	455 (24.5)	239 (24.8)	216 (24.2)
Secondary	504 (27.1)	256 (26.6)	248 (27.8)
University	898 (48.4)	469 (48.7)	429 (48.0)
**Dietary supplements**			
Vitamin D	28 (1.5)	12 (1.2)	16 (1.7)
Vitamin B complex	7 (0.4)	5 (0.5)	2 (0.2)
Vitamin C	11 (0.6)	5 (0.5)	6 (0.7)
Multivitamins	13 (0.7)	6 (0.6)	7 (0.8)
Multivitamins and minerals	27 (1.4)	15 (1.6)	12 (1.3)
Iron	7 (0.4)	5 (0.5)	2 (0.2)
Calcium	7 (0.4)	4 (0.4)	3 (0.3)
Fatty acids	14 (0.8)	6 (0.7)	8 (0.9)
Others	32 (1.7)	17 (1.8)	15 (1.7)
Total	131 (7.0)	63 (6.6)	68 (7.5)
**Dietary information**			
1st recall/record	1862 (100)	967 (100)	895 (100)
2nd recall/record	1780 (95.6)	924 (95.6)	856 (95.6)
**Energy intake (kcal/day)**			
6–12 months	1065 ± 157	1109 ± 161	1019 ± 138
1–3 years	1449 ± 210	1479 ± 214	1380 ± 173
4–8 years	1749 ± 211	1847 ± 211	1652 ± 157
9–13 years	1993 ± 254	2109 ± 229	1879 ± 225
14–17 years	2151 ± 436	2375 ± 406	1881 ± 295
**EI/BMR**			
6–12 months	2.22 ± 0.47	2.22 ± 0.43	2.21 ± 0.51
1–3 years	1.88 ± 0.50	1.87 ± 0.46	1.90 ± 0.55
4–8 years	1.62 ± 0.51	1.64 ± 0.50	1.59 ± 0.52
9–13 years	1.43 ± 0.46	1.41 ± 0.46	1.45 ± 0.45
14–17 years	1.30 ± 0.38	1.32 ± 0.38	1.27 ± 0.37

^a^ number of inhabitants; ^b^ ≤10 years of education; * *p* < 0.05, significant differences between sex groups. Abbreviations: ENALIA: Encuesta Nacional de ALimentación en Población Infantil y Adolescente de España (National Dietary Survey in Spanish Children and Adolescents); SD: Standard deviation; BMI: Body Mass Index; Z-BMI: *z*-score for BMI-for-age; EI/BMR: Observed Energy intake/Basal Metabolic Rate ratio.

**Table 2:** Usual intakes (from food and beverage sources only) of vitamins in Spanish children and adolescents and inadequate intakes.

	Boys							Girls						
	Mean	SD	Median (P5–P95)	EAR	<EAR (%)	UL	>UL (%)	Mean	SD	Median (P5–P95)	EAR	<EAR (%)	UL	>UL (%)
**Vitamin A (μg/day)**														
6–12 months	903	161	895 (653–1181)	500 *				849	80	845 (726–988)	500 *			
1–3 years	879	224	860 (547–1278)	210	0.0			860	209	836 (562–1240)	210	0.0		
4–8 years	884	273	845 (516–1387)	275	0.0			807	297	753 (432–1365)	275	0.1		
9–13 years	922	339	863 (493–1552)	445	2.7			947	327	891 (527–1553)	420	1.0		
14–17 years	1048	376	987 (553–1748)	630	10.1			799	322	738 (402–1406)	485	13.0		
**Vitamin D (μg/day)**														
6–12 months	3.3	1.4	3.1 (1.4–5.9)	10 *		38	0.0	3.0	1.5	2.8 (1.1–5.7)	10 *		38	0.0
1–3 years	2.6	1.4	2.3 (0.9–5.2)	10	99.8	63	0.0	2.3	1.0	2.1 (0.9–4.1)	10	100	63	0.0
4–8 years	2.2	0.7	2.1 (1.2–3.5)	10	100	75	0.0	2.1	1.4	1.8 (0.7–4.8)	10	99.7	75	0.0
9–13 years	2.8	1.1	2.6 (1.4–5.0)	10	100	100	0.0	2.9	1.2	2.7 (1.3–5.1)	10	100	100	0.0
14–17 years	4.3	0.7	4.2 (3.3–5.4)	10	100	100	0.0	2.1	0.5	2.1 (1.4–3.0)	10	100	100	0.0
**Vitamin E (mg TE ^a^/day)**														
6–12 months	9.7	2.6	9.6 (5.7–14.2)	5 *				9.0	2.8	8.8 (4.9–13.8)	5 *			
1–3 years	8.9	3.1	8.5 (4.7–14.7)	5	7.2	200	0.0	8.1	2.7	7.7 (4.5–13.0)	5	9.1	200	0.0
4–8 years	8.1	1.9	7.9 (5.2–11.6)	6	13.5	300	0.0	7.6	1.7	7.4 (5.1–10.6)	6	16.6	300	0.0
9–13 years	9.5	2.2	9.4 (6.2–13.5)	9	43.1	600	0.0	9.5	2.4	9.2 (6.0–13.8)	9	45.9	600	0.0
14–17 years	10.7	2.7	10.4 (6.7–15.6)	12	71.3	800	0.0	9.3	2.1	9.1 (6.2–13.1)	12	89.0	800	0.0
**Thiamin** **(mg/day)**														
6–12 months	0.7	0.2	0.7 (0.4–1.0)	0.3 *				0.6	0.2	0.6 (0.4–0.9)	0.3 *			
1–3 years	1.0	0.2	0.9 (0.7–1.2)	0.4	0.0			0.9	0.2	0.9 (0.7–1.3)	0.4	0.0		
4–8 years	1.3	0.2	1.2 (0.9–1.6)	0.5	0.0			1.1	0.2	1.0 (0.8–1.4)	0.5	0.0		
9–13 years	1.4	0.3	1.4 (1.1–1.9)	0.7	0.0			1.3	0.2	1.3 (1.0–1.8)	0.7	0.0		
14–17 years	1.7	0.5	1.7 (1.1–2.6)	1	2.1			1.2	0.3	1.2 (0.9–1.7)	0.9	8.2		
**Riboflavin** **(mg/day)**														
6–12 months	1.3	0.3	1.3 (0.8–1.8)	0.4 *				1.2	0.3	1.2 (0.6–1.7)	0.4 *			
1–3 years	1.6	0.3	1.6 (1.1–2.1)	0.4	0.0			1.6	0.3	1.6 (1.1–2.1)	0.4	0.0		
4–8 years	1.8	0.3	1.8 (1.3–2.3)	0.5	0.0			1.6	0.3	1.6 (1.2–2.1)	0.5	0.0		
9–13 years	1.9	0.4	1.9 (1.3–2.6)	0.8	0.0			1.8	0.4	1.7 (1.2–2.5)	0.8	0.0		
14–17 years	2.2	0.5	2.2 (1.5–3.2)	1.1	0.3			1.6	0.4	1.6 (1.1–2.4)	0.9	1.4		
**Niacin (mg Eq. Niacin/day)**														
6–12 months	16.6	4.0	16.3 (10.5–23.5)	4 *				15.2	3.7	15.0 (9.4–21.5)	4 *			
1–3 years	23.6	3.9	23.4 (17.7–30.4)	5	0.0			23.0	4.6	22.5 (16.3–31.1)	5	0.0		
4–8 years	30.4	5.2	30.0 (22.7–39.5)	6	0.0			25.9	4.0	25.6 (19.9–33.0)	6	0.0		
9–13 years	34.0	5.5	33.6 (25.8–43.7)	9	0.0			31.0	6.0	30.6 (21.9–41.5)	9	0.0		
14–17 years	40.6	6.2	40.2 (31.1–51.4)	12	0.0			31.8	5.8	31.4 (23.0–41.9)	11	0.0		
**Vitamin B_6_ (mg/day)**														
6–12 months	1.3	0.3	1.3 (0.8–1.9)	0.3 *				1.2	0.3	1.2 (0.7–1.8)	0.3 *			
1–3 years	1.6	0.3	1.5 (1.1–2.1)	0.4	0.0	30	0.0	1.6	0.4	1.5 (1.0–2.3)	0.4	0.0	30	0.0
4–8 years	1.9	0.3	1.9 (1.5–2.5)	0.5	0.0	40	0.0	1.7	0.4	1.6 (1.1–2.3)	0.5	0.0	40	0.0
9–13 years	2.0	0.4	2.0 (1.5–2.7)	0.8	0.0	60	0.0	1.9	0.5	1.9 (1.2–2.8)	0.8	0.0	60	0.0
14–17 years	2.5	0.5	2.5 (1.8–3.3)	1.1	0.0	80	0.0	1.8	0.4	1.8 (1.3–2.5)	1	0.4	80	0.0
**Vitamin B_12_ (μg/day)**														
6–12 months	1.7	0.5	1.6 (1.0–2.5)	0.5 *				1.5	0.5	1.4 (0.8–2.4)	0.5 *			
1–3 years	3.2	0.8	3.1 (2.2–4.7)	0.7	0.0			3.2	0.9	3.1 (2.0–4.9)	0.7	0.0		
4–8 years	4.3	0.9	4.2 (3.0–5.9)	1	0.0			4.2	0.9	4.0 (2.9–5.8)	1	0.0		
9–13 years	5.2	1.3	5.0 (3.4–7.5)	1.5	0.0			4.8	2.0	4.4 (2.6–8.6)	1.5	0.0		
14–17 years	6.1	0.9	6.1 (4.8–7.7)	2	0.0			4.5	1.2	4.3 (2.9–6.8)	2	0.1		
**Folate (μg DFE ^b^/day)**														
6–12 months	232	48.8	231 (155–316)	80 *				212.9	47.3	213 (134–290)	80 *			
1–3 years	214	51.8	209 (139–307)	120	1.4	300	6.1	210.4	48.7	207 (138–297)	120	1.6	300	4.4
4–8 years	222	35.8	219 (168–285)	160	2.6	400	0.0	215.7	46.0	211 (149–299)	160	9.7	400	0.1
9–13 years	255	64.7	247 (164–373)	250	51.9	600	0.0	250.0	65.0	242 (158–368)	250	55.0	600	0.0
14–17 years	303	74.9	294 (197–439)	330	68.1	800	0.0	242.7	61.2	236 (155–353)	330	91.3	800	0.0
**Vitamin C (mg/day)**														
6–12 months	135	21.9	134 (101–172)	50 *				130	18.7	129 (100–162)	50 *			
1–3 years	103	39.6	98 (49–176)	13	0.0	400	0.0	94	35.2	90 (43–157)	13	0.0	400	0.0
4–8 years	95	33.4	91 (49–157)	22	0.0	650	0.0	91	40.3	83 (39–166)	22	0.3	650	0.0
9–13 years	108	43.0	102 (49–188)	39	1.7	1200	0.0	107	39.9	102 (52–180)	39	1.2	1200	0.0
14–17 years	127	52.5	119 (56–225)	63	7.8	1800	0.0	112	41.4	107 (55–187)	56	5.4	1800	0.0

^a^ TE: alpha-tocopherol equivalents; ^b^ DFE: dietary folate equivalents; * Adequate intake (AI) and, therefore, the percentage of subjects with inadequate intakes have not been calculated. Abbreviations: EAR: Estimated average requirement; UL: Tolerable Upper Intake Levels.

**Table 3:** Usual intakes of minerals and trace elements (from food and beverage sources only) in Spanish children and adolescents and inadequate intakes.

	Boys							Girls						
	Mean	SD	Median (P5-P95)	EAR	<EAR (%)	UL	>UL (%)	Mean	SD	Median (P5-P95)	EAR	<EAR (%)	UL	>UL (%)
**Calcium (mg/day)**														
6–12 months	733	177.3	731 (444–1025)	260 *		1500	0.0	690	198.9	690 (363–1013)	260 *		1500	0.0
1–3 years	928	178.5	925 (639–1227)	500	0.8	2500	0.0	879	161.9	866 (633–1165)	500	0.4	2500	0.0
4–8 years	956	159.1	950 (707–1231)	800	16.0	2500	0.0	903	147.6	896 (674–1158)	800	25.0	2500	0.0
9–13 years	1025	212.5	1012 (699–1395)	1100	66.1	3000	0.0	959	167.7	949 (701–1251)	1100	80.6	3000	0.0
14–17 years	1147	264.4	1126 (751–1615)	1100	46.0	3000	0.0	860	195.9	853 (552–1195)	1100	88.6	3000	0.0
**Phosphorus (mg/day)**														
6–12 months	701	208.5	689 (379–1063)	275 *				657	218.3	641 (328–1042)	275 *			
1–3 years	1182	242.9	1170 (804–1601)	380	0.0	3000	0.0	1127	237.8	1106 (777–1550)	380	0.0	3000	0.0
4–8 years	1322	196.6	1311 (1019–1662)	405	0.0	3000	0.0	1211	136.8	1205 (996–1445)	405	0.0	3000	0.0
9–13 years	1441	205.7	1430 (1123–1797)	1055	2.1	4000	0.0	1299	184.4	1292 (1008–1614)	1055	8.7	4000	0.0
14–17 years	1639	239.1	1630 (1261–2046)	1055	0.4	4000	0.0	1273	208.0	1265 (944–1627)	1055	14.6	4000	0.0
**Iron (mg/day)**														
6–12 months	12.9	4.70	12.9 (5.1–20.7)	6.9	10.9	40	0.0	11.7	4.26	11.9 (4.4–18.4)	6.9	14.2	40	0.0
1–3 years	11.1	3.36	10.7 (6.4–17.2)	3	0.0	40	0.0	10.7	2.99	10.4 (6.3–16.1)	3	0.0	40	0.0
4–8 years	11.3	1.93	11.2 (8.4–14.7)	4.1	0.0	40	0.0	10.2	1.96	10.0 (7.4–13.7)	4.1	0.0	40	0.0
9–13 years	12.7	1.69	12.6 (10.1–15.6)	5.9	0.0	40	0.0	12.1	1.98	12.0 (9.2–15.6)	5.7	0.0	40	0.0
14–17 years	15.6	3.11	15.3 (10.9–21.1)	7.7	0.0	45	0.0	12.0	2.43	11.8 (8.3–16.3)	7.9	2.9	45	0.0
**Potassium (mg/day)**														
6–12 months	1782	401.0	1768 (1147–2465)	700 *				1652	351.3	1642 (1092–2247)	700 *			
1–3 years	2272	407.6	2263 (1618–2958)	3000 *				2187	374.1	2174 (1595–2824)	3000 *			
4–8 years	2647	213.1	2642 (2306–3007)	3800 *				2403	335.0	2389 (1875–2975)	3800 *			
9–13 years	2872	481.7	2846 (2126–3706)	4500 *				2590	463.9	2568 (1867–3389)	4500 *			
14–17 years	3270	610.9	3233 (2333–4335)	4700 *				2590	494.8	2565 (1822–3445)	4700 *			
**Sodium (mg/day)**														
6–12 months	394	164.8	361 (197–706)	370 *				394	130.2	373 (224–637)	370 *			
1–3 years	1246	338.2	1203 (775–1865)	1000 *		1500	20.4	1158	323.9	1112 (719–1756)	1000 *		1500	13.9
4–8 years	1858	362.4	1820 (1333–2509)	1200 *		1900	41.5	1825	361.1	1781 (1317–2481)	1200 *		1900	37.8
9–13 years	2519	577.5	2447 (1710–3572)	1500 *		2200	68.4	2177	571.7	2092 (1411–3237)	1500 *		2200	42.3
14–17 years	2911	857.4	2785 (1756–4497)	1500 *		2300	75.1	2307	325.4	2285 (1812–2876)	1500 *		2300	49.0
**Zinc (mg/day)**														
6–12 months	6.2	1.73	6.3 (3.3–9.0)	2.5	1.5	5	76.0	5.7	1.67	5.8 (2.9–8.4)	2.5	2.8	5	67.3
1–3 years	7.5	1.46	7.4 (5.3–10.1)	2.5	0.0	7	61.2	7.3	1.67	7.2 (4.9–10.3)	2.5	0.0	7	54.8
4–8 years	8.8	1.45	8.6 (6.7–11.4)	4	0.0	12	2.6	7.9	1.08	7.8 (6.3–9.8)	4	0.0	12	0.2
9–13 years	9.8	0.98	9.7 (8.3–11.5)	7	0.0	23	0.0	8.7	1.38	8.6 (6.6–11.2)	7	9.2	23	0.0
14–17 years	11.3	1.50	11.2 (9.0–13.9)	8.5	2.1	34	0.0	8.9	1.20	8.9 (7.1–11.0)	7.3	6.3	34	0.0
**Magnesium (mg/day)**														
6–12 months	123.0	28.3	121 (79–172)	75 *				112.6	21.9	112 (78–150)	75 *			
1–3 years	196.7	38.9	195 (135–263)	65	0.0			190.2	36.3	188 (134–253)	65	0.0		
4–8 years	248.1	27.4	247 (205–295)	110	0.0			228.0	18.4	227 (199–259)	110	0.0		
9–13 years	280.1	39.9	278 (218–349)	200	1.4			255.8	32.7	254 (205–312)	200	3.4		
14–17 years	321.7	66.4	317 (222–439)	340	63.5			256.7	48.5	254 (182–341)	300	81.8		
**Selenium (μg/day)**														
6–12 months	25.7	11.4	23.7 (10.9–47.3)	20 *		60	1.1	22.5	7.9	21.7 (10.9–36.6)	20 *		60	0.0
1–3 years	60.2	15.9	58.9 (36.5–88.4)	17	0.0	90	4.2	58.8	21.4	55.9 (29.5–98.2)	17	0.2	90	8.4
4–8 years	90.4	20.1	88.3 (61.2–126.5)	23	0.0	150	0.8	81.0	10.5	80.4 (64.7–99.2)	23	0.0	150	0.0
9–13 years	109.1	14.2	108.3 (87.1–133.7)	35	0.0	280	0.0	95.8	11.1	95.3 (78.6–114.9)	35	0.0	280	0.0
14–17 years	131.3	24.0	129.8 (94.5–173.2)	45	0.0	400	0.0	105.3	19.5	103.8 (75.8–139.7)	45	0.0	400	0.0
**Iodine (μg/day)**														
6–12 months	76.8	13.0	76.5 (56.2–98.8)	130 *				69.6	13.8	69.8 (46.7–92.1)	130 *			
1–3 years	84.6	17.0	83.2 (59.3–114.5)	65	11.0	200	0.0	85.5	15.4	84.5 (62.1–112.3)	65	7.8	200	0.0
4–8 years	87.3	16.1	85.6 (64.1–116.1)	65	5.9	300	0.0	84.1	13.3	82.8 (64.9–107.9)	65	5.1	300	0.0
9–13 years	104.3	15.0	103.0 (82.0–131.0)	73	0.6	600	0.0	86.5	16.0	84.6 (63.9–115.6)	73	19.8	600	0.0
14–17 years	107.2	22.9	104.6 (74.6–148.5)	95	31.9	900	0.0	91.2	13.3	90.1 (71.4–114.8)	95	64.3	900	0.0

* Adequate intake (AI) and, therefore, the percentage of subjects with inadequate intakes have not been calculated.
